# Engaged curiosity: “Hot spots” for clinical reasoning in complex patient encounters

**DOI:** 10.1016/j.pecinn.2025.100408

**Published:** 2025-06-06

**Authors:** Michael Soh, Dolores Mullikin, Steven J. Durning, Jerusalem Merkebu

**Affiliations:** a School of Graduate Studies, University of Maryland, Baltimore, USA; b Mary Bridge Children's Hospital, Tacoma, WA, USA; c Department of Health Professions Education, Uniformed Services University, USA

**Keywords:** Clinical reasoning, Engaged curiosity, Empathic concern, Medical education

## Abstract

**Objective:**

This study explores how, if at all, engaged curiosity - a genuine, emotionally engaged interest in learning more about the complexity of another's particular emotional perspective - emerges in the clinical reasoning process and its relationship with contextual factors and clinical reasoning performance.

**Methods:**

Think-alouds transcripts from nineteen physicians in internal medicine from three military training facilities were thematically analyzed for instances of engaged curiosity and examined through the lens of contextual factors and clinical reasoning performance.

**Results:**

Our findings indicate that engaged curiosity can be likened to placeholders that physicians employ early on to “bookmark” sources of patient concern. These sources, or hot spots, deserve follow up, particularly when cognitive resources are unavailable to “attend” to a deeper understanding of the patient's experience.

**Conclusion:**

Engaged curiosity provides a unique lens for better understanding the relationship between empathy and clinical reasoning and warrants further research on its impact on the patient and their care.

**Innovation:**

Engaged curiosity could serve as a novel way to train physicians to think and engage more empathically with their patients.

## Highlights

• Engaged curiosity—a physicians' emotional interest in patient concerns—has been identified in the clinical reasoning process
• Engaged curiosity acts as a "bookmark" for sources of patient concern, or hot spots, that deserve follow-up during patient encounters
• Future research should explore if engaged curiosity serves as a proxy for physician empathy, or a step toward empathic concern

## 1 Introduction

Diagnostic error is the most prevalent, costly, and devastating cause of preventable medical harm [1]. In fact, it was estimated that diagnostic error is the third leading cause of death in the United States and is responsible for approximately 10 % of patient deaths and hospital adverse events [2]. Existing literature on clinical reasoning (defined as the cognitive steps up to and including arriving at a diagnosis and management plan for a patient) suggests that the specifics of the clinical situation (referred to as context specificity and associated contextual factors) can contribute to these harmful errors [[3], [4], [5]].

Context specificity refers to the phenomenon of a physician seeing two patients with the same symptoms and findings, and ultimately the same diagnosis, but yet comes to two different diagnostic decisions [3,4]. In other words, something beyond the content (knowledge) of the patient's presentation is influencing decision making. Recently, scholars have used the lens of situated cognition theory, which argues that cognitive tasks, such as clinical reasoning, are bound to the situations in which they occur [3,4]. These situational factors, which we refer to as contextual factors*,* arise from patients, physicians, and environment and can include diagnostic suggestions, interruptions, language barriers, physician fatigue, and emotion. They can negatively affect clinical reasoning and can lead to incorrect diagnosis and resultant incorrect treatment, even across levels of expertise and training [3,6].

Part and parcel to this situated context is the role and impact of emotion and more specifically, the empathic nature of the physician during the patient encounter. Research has shown that a physician's empathy may play a critical role in the clinical reasoning process. Studies have shown that empathic physician-patient interactions can lead to more accurate diagnoses and prognoses [[7], [8], [9]]. As a result of an empathic physician, patients will reveal more about their medical concerns and allow for more detailed information gathering about a patient's symptoms, history, and individual needs [7,[10], [11], [12], [13]]. Additionally, empathy can lead to an increase in the physician's understanding of patients' perspectives, which therein leads to improvements in patients' perceptions of being helped, patients' perceptions of a support network, and patients' empowerment [14,15]. The multiple and varied ways empathic interactions impact the patient as well as the physician's clinical reasoning process simply underscore how complex the clinical encounter can be.

Yet, despite this clear demonstration of how empathy can positively impact patient care, existing scholarship remains complex and nuanced on the types of empathy and perhaps more importantly, if, how and when they unfold in the clinical setting. Whether it be cognitive empathy, affective empathy, clinical empathy, or something in between, scholars have debated how and when to deploy the various dimensions of empathy available in the literature. Thus, given the context of this study, we found it imperative that we rely on a construct that factored in the complexities of clinical reasoning and more generally, patient care. As such, we relied on Halpern's proposed construct of *engaged curiosity* [16,17]. Halpern describes engaged curiosity as a “genuine, emotionally engaged interest in learning more about the complexity of the patient's (and the physician's responsive) emotional points of view about another's particular emotional perspective”. This stance recognizes that the physician does not “fully understand and has more to learn about the patient's situated experience - what the patient is afraid of, needing or seeking” [17], [p. 305, 308]. Most importantly, Halpern argues that engaged curiosity is particularly important in complex patient encounters when emotions and distress may be at a higher level [17]. Guidi and Traversa [18] extend Halpern's work further by arguing that engaged curiosity may be a predisposition to *empathic concern* - a re-conceptualization of empathy that aims to synthesize and highlight the essences of cognitive, affective, and clinical empathy. In short, engaged curiosity seems to capture an earlier signal, or predisposition, of what empathy might look like in the midst of contextual factors while more importantly, also serving as a marker, or precursor, for the physician to move beyond mere interest in learning more, and into fully understanding the patient's situated experience. Improved understanding of the patient's situated experience will then help the physician with their clinical reasoning, allowing for a management plan that aligns with the patient's situated experience.

Therefore, by better understanding if and how engaged curiosity unfolds in the clinical reasoning process and how it impacts performance, we can begin identifying subsequent empathic strategies and techniques that might help minimize diagnostic error and improve diagnosis and management, particularly in the presence of contextual factors. Hence, our study begins to explore both engaged curiosity and clinical reasoning, particularly in the presence and absence of contextual factors, and aims to answer the following research questions:

1. How does engaged curiosity emerge in the clinical reasoning process, if at all?
2. What relationship, if any, do contextual factors have with engaged curiosity?
3. What relationship, if any, does engaged curiosity have with clinical reasoning performance?

## 2 Methods

This study relied on existing data from a previous study on clinical reasoning and context specificity [6]. The sample included 19 physicians in internal medicine recruited from the Uniformed Services University of the Health Sciences, Walter Reed National Military Medical Center, and Naval Medical Center San Diego. Approval was received from all Institutional Review Boards and complied with the World Medical Association Declaration of Helsinki.

Briefly, in said previous study [6], both live and video simulation cases were designed, depicting patients with typical presentations of common diseases in primary care practice: new-onset diabetes mellitus and unstable angina. Participants in the live cases participated in the case as a physician rather than viewing a video of another physician in a previously recorded patient encounter (resulting in a final sample size of 19 physicians). Each case included a clinical interview, a brief physical exam, and still screens of laboratory findings. A group of internal medicine physicians tasked with designing said cases chose commonly encountered cases and contextual factors to mitigate the effects of case (i.e. content) specificity [19] and enhance ecological validity. Thus, study participants participated in one of two conditions: (a) diabetes case with inhibiting contextual factors (low English proficiency and a patient questioning the physician's credentials); angina case without contextual factors, or (b) angina case with inhibiting contextual factors (misleading diagnostic suggestion, patient reports history circuitously); diabetes case without contextual factors.

After completing their assigned simulation cases, participants completed a post-encounter form (PEF) as a measure of clinical reasoning performance. The PEF asks for: (1) additional information they would like to obtain by history, (2) additional physical exam actions they would take, (3) a problem list, (4) a differential diagnosis, (5) a leading diagnosis, (6) supporting evidence for the diagnosis, and (7) management plans. Participants were given up to 30 min (determined to be ample time in prior trials) for completing the items. Items were scored as in prior research where reliability and validity evidence for this instrument were established [4,[20], [21], [22], [23]].

After completing each PEF, participants completed a standard think-aloud procedure. More specifically, participants re-watched a recording of each of their own simulated cases while “thinking aloud” with the task of establishing a diagnosis and treatment plan for the patient portrayed in the videotape. In this procedure, participants verbalize whatever thoughts they have during a task and are explicitly instructed to not analyze their thought processes. Most importantly, think-alouds have been shown in several studies to reliably reflect concurrent mental processes while performing a task [24]. Additionally, prior think-aloud research involving physicians revealed a discernible transition in cognitive focus, aligning more closely with patient perspectives, providing valuable insights into their interactions [6]. All think-alouds were recorded.

Think-alouds were blinded, i.e. coders were unaware of which think-alouds accounted for a contextual factor prior to analysis, and then deductively coded for engaged curiosity. Our operationalization of engaged curiosity was grounded by the previously mentioned definition put forth by Halpern [16,17]. We then extended our understanding of engaged curiosity by integrating Guidi and Traversa's notion that engaged curiosity may be a “validation of the patient's suffering and needs” that “allows patients to feel recognized, understood, and accepted in their hardship”. Furthermore, Guidi and Traversa [18,p 508] argue that this type of empathic engagement is “reached through curiosity about what the patient is specifically concerned about, non-verbal attunement and the effort to imagine the other's experience” and that the physician is then “led to welcome a deeper and more comprehensive understanding of the patient's experience”. Thus, our deductive coding schema for engaged curiosity relied on several criteria: 1) temporality, i.e. only the first sign of an emotionally-engaged interest in “learning more” about the patient's condition was coded, 2) continuity, i.e. subsequent thoughts and deliberation build upon the initial desire to “learn more”, 3) patient activated, i.e. emotionally-engaged interest was triggered by the patient, and 4) empathically oriented, i.e. emotions such as concern or worry about the patient were tied to the emotionally-engaged interest.

All transcripts were coded by MS and JM, both health professions educators with expertise in both quantitative and qualitative research methodologies and an interest in emotions, clinical reasoning, and promoting patient-centered care. Due to the flexibility of thematic analysis, the aforementioned approach allowed for exploration of trends and patterns of engaged curiosity during the clinical reasoning process [25]. As Fereday and Muir-Cochrane suggest, the interaction of text, codes, and engaged curiosity themes involved several iterations of interpretive analysis until consensus was reached. For example, several instances of “emotionally engaged interest in learning more” were excluded from analysis due to irrelevance to the perceived trajectory of the clinical reasoning process, the original domain of inquiry for this work. Non-parametric statistical analyses were also used to explore the relationships between engaged curiosity, context specificity, and clinical reasoning performance and are reported below.

## 3 Results

### 3.1 Emergence of engaged curiosity

In our analysis of 38 think-aloud transcripts from the 19 participants, forty-four instances of engaged curiosity were identified. These 44 moments of engaged curiosity were scattered across 20 think-aloud transcripts (16 with a contextual factor, 4 without) that represented 16 unique participants. Ultimately, 18 think-aloud transcripts were not included for analysis because they did not contain any codes for engaged curiosity. Of the 16 participants, seven were interns, three were residents, and six were attending faculty. Engaged curiosity stances ranged in frequency from 1 per think-aloud transcript up to 7 in a given case transcript.

In alignment with Halpern's [17] definition of engaged curiosity, all identified codes represented the first sign of “genuine, emotionally engaged interest” from the physician and were activated by the patients themselves via conversation. The patient's thoughts, feelings, and experiences - rooted in the respective simulated conditions - triggered emotions that seem to point in the general direction of concern, worry, and at times, alarm, and led to continued deliberation or thought related to the original source of interest. The physicians' heightened vigilance inherently embeds the patient's emotional perspective within their presentations, as illustrated by examples of ‘worry’ about intervention and negative intuitive responses (“hackles go up”) to functional limitations. The following examples demonstrate an awareness of the broader impact of illness and emotional sensitivity to patient distress. (see Table 1 for additional examples):

**Table 1:** Example quotes of engaged curiosity from think-aloud transcripts.

Unique ID	Engaged curiosity
JPC068R	*“…immediately I'm concerned for.. um.. anginal chest pain or typical chest pain.”*
JPC065	*“The tightness or pressure is something that I worry about with regard to ischemic disease.”*
JPC038	*“I would be concerned about heart disease until proven otherwise.”*
JPC014	*“The diabetes and hypertension seems to kind of run in his family as well. Both of his parents are living, so that's… good. Yeah, so that's another red flag there for me.”*
JPC036	*“Just all these alarm bells are going off in my head about, ding, ding, ding, ding, ding.”*
JPC047	*“I don't know, these are thing things that's really, to me, is concerning for a cardiac origin.”*
JPC022	*“…so when you have those all together, you worry about them together, but obviously…”*
JPC-I-006	*“In my mind, I was already, like, alright, heart concern…”*

*“…but I'm worried, at this point, that if he's got something*

*going on that we are gonna not only find something,*

*but he might need more intervention than just a stress test…”*

*[Unique ID: JPC022]*

*“…my hackles just go up when I hear people who get more winded,*

*get exercise intolerance. It's like a big red flag.*

*That makes me more concerned, too.”*

*[Unique ID: JPC036]*

*“…my concern is always– most highest concern is for any kind of*

*angina, anginal symptoms, any kind of cardiac history.”*

*[Unique ID: JPC-I-008]*

### 3.2 Engaged curiosity and context specificity

Physicians in contextual factor cases demonstrated 38 instances of engaged curiosity while physicians in non-contextual factor cases demonstrated six instances of engaged curiosity. A Kruskal-Wallis test showed that there was a statistically significant difference in the frequency of engaged curiosity codes between contextual and non-contextual factor cases, χ^2^(1) = 14.984, *p* < 0.001.

Engaged curiosity was also temporally exhibited differently by physicians in a contextual or non-contextual factor case. For example, on average, the *first* engaged curiosity code in a contextual factor case transcript emerged at word count 341. In comparison, on average, the *first* engaged curiosity code in a non-contextual factor case transcript emerged as word count 708. A Mann-Whitney *U* test showed a statistically significant difference in these initial appearances when comparing contextual and non-contextual factor cases [U = 10.00, *p* = 0.039].

### 3.3 Engaged curiosity and clinical performance

Mann-Whitney *U* tests showed no significant differences in PEF scores between those who demonstrated engaged curiosity versus those who did not. However, notable trends were observed in the PEF scores between said two groups. For example, scores trended higher for those who exhibited engaged curiosity when it came to reporting additional exam actions they would take, their problem list, their differential diagnoses, and their leading diagnosis. In contrast, scores trended lower for those who exhibited engaged curiosity when it came to reporting additional questions they would ask, their supporting data for their leading diagnosis, and their treatment plan.

## 4 Discussion and conclusion

### 4.1 Discussion

The purpose of this study was to explore if and how engaged curiosity emerged in the clinical reasoning process and whether any relationship existed between engaged curiosity, context specificity, and clinical performance. Our findings reveal engaged curiosity does in fact emerge in the clinical reasoning process, as demonstrated by our analysis of think-aloud data. Interestingly, engaged curiosity seems to be more prevalent in the presence of contextual factors, which provides evidence for Halpern's earlier claim that engaged curiosity may be especially important in complex patient encounters when emotions and distress are at a higher level. In fact, in the absence of constraining contextual factors, the presence of engaged curiosity is quite minimal. It is plausible, engaged curiosity can be likened to a placeholder, or perhaps a red flag, that physicians employ early on to “bookmark” sources of patient concern, or *hot spots*, that appropriately deserve follow up; particularly, in the presence of contextual factors, when cognitive resources are unavailable to “attend” to deeper and more comprehensive understanding of the patient's experience [3]. Social psychology research has delineated *hot spots* as areas of leaked clues to attend to in stress inducing environments [26]. Engaged curiosity also tends to appear earlier in a patient encounter with a contextual factor versus not. Based on this temporal analysis, physicians may be leaning in on these *hot spots* – “bookmarking” sooner when distractors are readily apparent. A better understanding of the follow up process beyond these initial instances of engaged curiosity and why they tend to occur earlier in more complex encounters deserves further study, particularly in determining if and how empathic concern might emerge in the clinical reasoning process.

We also found no significant differences in clinical reasoning performance between those who exhibited engaged curiosity and those who did not. Although the literature has indicated that physician empathy during the patient encounter leads to higher diagnostic accuracy, it is unclear if engaged curiosity and empathy are one and the same. On one hand, as Guidi and Traversa argued [18], engaged curiosity may be a precondition for empathic concern, or a reconceptualization of clinical empathy. If so, there may be additional “steps” in the empathic cycle, if you will, that need to be completed, or unlocked, in order to achieve higher diagnostic accuracy. On the other hand, one could argue that engaged curiosity may serve as a proxy, or substitute, for empathy, and that engaged curiosity may be sufficient, or even preferred, by patients. Recent research has indicated that what patients desire as empathic behaviors from their physicians largely varies [27], and because no consensus exists, engaged curiosity could represent yet another empathic behavior that physicians can deploy with their patients.

Engaged curiosity is especially noteworthy because it may also lead to empathic distress. Singer and Klimecki ([28], p R875) define empathic distress as a “strong aversive and self-oriented response to the suffering of others, accompanied by the desire to withdraw from a situation in order to protect oneself from excessive negative feelings”. Withdrawing from a patient, particularly during a complex case, can hinder the gathering of much needed information and context and thus, impair diagnostic accuracy. Thus, a better understanding of when and how engaged curiosity manifests in the clinical reasoning process could help identify if, when, and how empathic distress might derail the clinical reasoning process.

Our findings may also indicate that engaged curiosity could be a protective factor in clinical reasoning performance. That is, given that there were no significant differences in post-encounter form scores in contextual versus non-contextual factor cases, engaged curiosity may be down regulating any potential negative impact of contextual factors on clinical reasoning performance. “Bookmarking” areas of patient concern may be helping physicians flexibly jump to different tasks in the clinical reasoning process that demand their more immediate attention [29,30]. This line of inquiry also deserves further study.

This study had several limitations. First, we relied on secondary data that implemented a study design that may not have been ideal for promoting empathic reactions, e.g. simulated scenarios with standardized patients. While we recognize the uniqueness of this particular data, future studies may want to consider shaping cases that more strongly trigger empathic emotions, or studying engaged curiosity in a more ‘authentic’ interaction in clinical practice. As Halpern [17] noted, engaged curiosity may play a bigger role in complex cases when emotions and distress may be at a higher level. In other words, the simulated nature in which our secondary data was collected may have prevented, or hindered, empathic reactions to fully develop. Our study may be worth replicating using more tailored cases, with a larger, more diverse sample. Second, given the sample size, we could not appropriately account for the impact of training level, i.e. intern, resident, and attending. We suspect that experience plays a role in the demonstration of engaged curiosity and future work may want to focus on this element during study recruitment. And third, and perhaps most importantly, it remains unclear if, and when, engaged curiosity becomes empathic concern. Though we allude to the temporality of engaged curiosity and empathic concern in our study rationale, because think-alouds only capture the physician perspective, we were unable to specifically confirm if a patient's needs or suffering were being *validated*. Future research may want to explore these two constructs further, along with incorporation of the patient voice.

### 4.2 Innovation

Engaged curiosity is an entity, as we have described, that fluctuates based upon the clinical scenario and may represent an intentional “tool” that physicians can turn to when presented with a complex patient. For instance, a practicing physician can pause in the room and ask themselves an intentional question to spur engaged curiosity, such as “do I understand the patient's point of view”? This simple question may then lead to further questioning that results in more details shared with the physician, better clinical reasoning, a stronger patient-physician relationship, and culturally responsive trauma informed care.

Framing the ideas of engaged curiosity into an entity may allow it to be taught in medical education as a clinical reasoning strategy that could enhance patient centered care. Engaged curiosity may serve as a prescribed first step for establishing beneficial patient physician interactions, improved clinical reasoning and protection from burnout. In fact, it is entirely possible that training physicians to have engaged curiosity may yield many of the benefits attributed to “empathy”. These “empathic opportunities” as noted by Frankel [31 ,p 89] may represent yet another dimension of the hidden curriculum of medical education that if made more transparent could improve training practices.

### 4.3 Conclusion

Engaged curiosity may provide a unique lens for how we understand the relationship between empathy and clinical reasoning. Our study describes engaged curiosity in the context of clinical reasoning and situated cognition and its potential role in the larger conversation about the role of empathy in patient care. The very strategy of expressing an emotionally-engaged desire to learn more about a patient's concern may not only have benefits for clinical reasoning, but patient-centered care as a whole. Particularly during patient encounters where emotions and distress run high, engaged curiosity could serve as a novel tool for training physicians to think and engage more empathically with their patients.

## CRediT authorship contribution statement

**Michael Soh:** Writing – review & editing, Writing – original draft, Visualization, Validation, Supervision, Software, Resources, Project administration, Methodology, Investigation, Formal analysis, Data curation, Conceptualization. **Dolores Mullikin:** Writing – review & editing, Writing – original draft, Validation. **Steven J. Durning:** Writing – review & editing, Resources, Project administration, Funding acquisition, Data curation. **Jerusalem Merkebu:** Writing – review & editing, Validation, Project administration, Methodology, Formal analysis.

## Declaration of competing interest

The authors declare the following financial interests/personal relationships which may be considered as potential competing interests:

Michael Soh reports article publishing charges was provided by American Educational Research Association. If there are other authors, they declare that they have no known competing financial interests or personal relationships that could have appeared to influence the work reported in this paper.
